# Abstracts of the 2023 Canadian Association of Medical Oncologists Annual Scientific Meeting †

**DOI:** 10.3390/curroncol30080550

**Published:** 2023-08-11

**Authors:** Alexi Campbell, Sharlene Gill, Desiree Hao, Erin Powell, Stephanie Snow, Zachary Veitch, Stephen Welch

**Affiliations:** Canadian Association of Medical Oncologists, Ottawa, ON K1E 3R9, Canada; sgill@bccancer.bc.ca (S.G.);

**Keywords:** medical oncology, cancer, research

## Abstract

On behalf of the Canadian Association of Medical Oncologists, we are pleased to present the abstracts of the 2023 Annual Scientific Meeting. The CAMO Annual Scientific Meeting (ASM) took place on 27 April 2023 in an in-person event in Toronto, ON. Thirty-two (32) abstracts were selected for presentation as oral presentations, in-person poster presentations, and virtual poster presentations. Awards for the top four (4) abstracts were presented during the ASM; they have been marked as “Award Recipient”. We congratulate all presenters on their research work and contribution.

## 1. Oral Presentation

**Mainstream Model of Genetic Testing for Prostate Cancer: The Sunnybrook Odette Cancer Centre Experience**
Xin Wang ^1,2,3^, Caleb Tackey ^3^, Larissa Waldman ^4^, Yael Silberman ^4^, Danny Vesprini ^5^, Urban Emmenegger ^2,3^, Andrea Eisen ^2,3,4^ and Martin Smoragiewicz ^2,3^

1. Department of Medical Oncology, Princess Margaret Cancer Centre, Toronto, ON
2. Department of Medical Oncology, Sunnybrook Odette Cancer Centre, Toronto, ON
3. Temerty Faculty of Medicine, University of Toronto, Toronto, ON
4. Cancer Genetics and High Risk Program, Sunnybrook Odette Cancer Centre, Toronto, ON
5. Department of Radiation Oncology, Sunnybrook Odette Cancer Centre, Toronto, ON

**Background**

An estimated 20–30% of men with prostate cancer carry a mutation in DNA damage repair genes. The eligibility criteria for germline genetic testing has significantly expanded, and many centres have adopted a “mainstreaming” model, which is defined as oncologist-initiated genetic testing.

**Methods**

Between 1 May 2021 and 30 May 2022, 174 eligible patients with prostate cancer underwent mainstream testing using a 19-gene panel. Descriptive and inferential statistics were used to compare patients with and without a germline mutation.

**Results**

The median age was 75 years (IQR 68.25–80 years), and 72% of patients were diagnosed with either de novo metastatic or high-risk localized prostate adenocarcinoma. Fourteen patients (8%; 95% CI 4–12%) were found to have a deleterious germline mutation, including likely pathogenic mutations in *BRCA1/2*, *ATM*, *CHEK2*, *PMS2*, *RAD51C*, *HOXB13*, and *BRIP1*. Forty-nine patients (28%; 95% CI 21–35%) were found to have a variant of unknown significance. Patients with germline mutation were not statistically different from those without a mutation in terms of baseline clinico-demographic features, including age, stage at diagnosis, baseline PSA, and prior lines of treatment. Thirty-four patients within our cohort also received panel-based NGS testing of their somatic tissue. Among this subset, 23% (8 of 34) had an alteration in homologous recombination-related genes, as was defined per PROfound trial. Of the 14 patients with germline mutation, none had a prior personal history of malignancy, and 6 (43%) did not have any first- or second-degree relatives with a history of prostate, pancreatic, or breast cancer. The median turnaround time for genetic results was 91 days (IQR 37–113 days).

**Conclusion** **s**

We demonstrated the feasibility of a mainstream model for germline genetic testing in prostate cancer patients. Personal history and family history of cancer cannot reliably stratify patents for the presence of pathogenic germline variants. Further work is needed to demonstrate clinical utility.

**Award Recipient**

## 2. Oral Presentation

**Does Community Size Impact Survival with Breast Cancer? Data from a Large Population-Based Cohort in British Columbia**
Emily Jackson ^1^, Lovedeep Gondara ^2^, Caroline Speers ^2^, Rekha Diocee ^2^, Alan Nichol ^3^, Caroline Lohrisch ^1^, Stephen Chia ^1^

1. BC Cancer, Department of Medical Oncology, Vancouver, BC
2. Breast Cancer Outcomes Unit, BC Cancer, Vancouver, BC
3. BC Cancer, Department of Radiation Oncology, Vancouver, BC

**Objective**

The purpose of this study is both a descriptive and quantitative analysis of the impact of rural versus urban residency on baseline clinical-pathological features and outcomes for individuals diagnosed with breast cancer in British Columbia.

**Methods**

Using the BC Cancer’s Breast Cancer Outcomes Unit database, we identified all patients with invasive breast cancer that were diagnosed from 2005 to 2018 and categorized the patients as residing in either an urban (population ≥ 100,000 people) or rural setting (< 100,000 people) using postal code. We analyzed the baseline features, initial treatment, and outcomes differences between these settings.

We performed a univariable analysis examining differences in locoregional relapse, distant relapse, and breast cancer-specific survival (BCSS). We performed a multivariable analysis accounting for age, grade, lymphovascular invasion (LVI), subtype, stage, chemotherapy, endocrine therapy (ET), radiotherapy (RT), and type of definitive surgery.

**Results**

The study included 35,255 patients. There were no clinically meaningful differences in the baseline characteristics. Rural patients were significantly more likely to undergo mastectomy (43.4% vs. 39.1%) and were less likely to receive RT (61.4% vs. 67.7%) or ET (67.2% vs. 71.7%). There was no difference in the use of chemotherapy or anti-HER2 directed therapies.

A univariable analysis revealed an inferior BCSS in the rural cohort (85.3% [95% CI:84.5–86.1%] vs. 86.5% [86.0–86.9%], *p* < 0.001). Risk of distant relapse was higher in the rural setting (13.4% [12.6–14.1%]) compared with the urban setting (12.1% [11.7–12.6%], *p* < 0.001). There was no difference in locoregional relapse.

In the multivariable analysis, urban residency was shown to improve BCSS, featuring a hazard ratio (HR) of 0.92 [0.86–0.99, *p* = 0.03]. Urban residency was also associated with a lower distant relapse rate (HR = 0.91 [0.85–0.98, *p* = 0.01]). There was no impact on locoregional relapse.

**Conclusions**

Rural residency is a significant and independent risk factor for both distant relapse and mortality from breast cancer. Understanding the factors that contribute to this disparity is necessary to close the gap between rural and urban breast cancer outcomes.

## 3. Oral Presentation

**A Comprehensive Framework For Assessing and Improving Wellness in the Medical Oncology Training Program at the University Of Ottawa**
Julian Surujballi and Paul Wheatley-Price
The Ottawa Hospital Cancer Centre, University of Ottawa, Ottawa, ON, Canada

**Objective**

Wellness is a growing area of concern among physicians and residents. This quality improvement project aims to assess and improve resident wellness in the Medical Oncology Training Program at the University of Ottawa.

**Methods**

A focus group was constructed between residents and the training program administrator to identify all tasks related to residency training. Residents rated the time spent, frequency, perceived learning benefit, and wellness detriment for each task on a five-point Likert scale and provided narrative comments. Medical Oncology staff were independently asked to rate tasks for their learning benefit only. Tasks were flagged for concern if they were rated as both high wellness detriment and low learning benefit, if learning benefit ratings significantly differed between staff and residents, or if they were directly flagged by the residents.

**Results**

The study identified 38 unique tasks. Residents spent an average of 91.0 h/week on training-related tasks, which rose to 101.2 h/week for chief residents. Non-chief residents spent 66.9 h/week on mandatory activities and 24.1 h/week on non-essential activities. Residents spent approximately 49% of their time on clinical duties, 20% on professional development, 12% on service, and 8% on administrative duties; the remainder was split between attending rounds, completing evaluations, and academic time. Three activities yielded higher than expected time commitments: research (10 h/week), mock oral exams (8 h per exam), and preparing consultations before clinic appointments (1 h per consult or 7.5 h/week).

Process adjustments were suggested for all 17 items that were flagged for concern. In addition to reducing the unnecessary wellness detriment, these changes could decrease the average working time to 73.9 h/week for non-chief residents.

**Conclusions**

This project provides a framework for identifying training- or work-related tasks that are excessively detrimental to wellness compared with their benefit. This process can be repeated to assess the effectiveness of change and can be replicated for other groups such as staff physicians or nurses.

## 4. Oral Presentation

**Benefit of Adjuvant Bisphosphonates in Early Breast Cancer Treated with Contemporary Systemic Therapy: A Meta-Analysis of Randomized Control Trials**
Abhenil Mittal, Faris Tamimi, Consolacion Molto Valiente, Massimo Di Iorio and Eitan Amir
Division of Medical Oncology and Hematology, Princess Margaret Cancer Center, Toronto, ON

**Background**

The benefit of adjuvant bisphosphonates (BPs) in patients receiving contemporary systemic therapy remains uncertain. Therefore, we performed this meta-analysis to determine the absolute and relative benefits of adjuvant BPs on DFS and OS in patients with early-stage breast cancer in contemporary trials.

**Methods**

Using references from the American Society of Clinical Oncology guidelines on the use of adjuvant BPs (2017 and 2022), we selected randomized trials that recruited patients exclusively after 2000 and extracted the 5-year DFS and OS in the BP and control group arm along with associated hazard ratios (HRs). DFS and OS data were weighed by the study sample size. The HRs for DFS and OS were pooled in a meta-analysis using generic inverse variance and random effects modelling. A meta-regression comprising linear regression weighted by sample size (mixed effects) was performed to explore the association between disease- and treatment-related factors and the absolute differences in the benefit resulting from BPs.

**Results**

The analysis included 11 trials comprising 24,023 patients. For DFS, the pooled HR across trials was 0.89 (0.81–0.97, *p* = 0.008) with a 1.5% weighted mean difference that favored BPs over the control. There was no significant OS benefit with BPs (HR 0.92, 0.82–1.03, *p* = 0.16). Among patients receiving anthracycline- and taxane-based chemotherapy, there were no differences in either DFS (HR 0.95, 95% CI 0.80–1.12) or OS (HR 1.04, 95% CI 0.81–1.32). The meta-regression results showed that DFS and OS benefit in higher-risk patients (node-positive, larger tumor size, ER-, grade 3, or those receiving chemotherapy) was lower. Overall, 1% (95% CI 0.75–1.15) of patients experienced ONJ related to zoledronic acid.

**Conclusions**

Compared with the data reported by the Early Breast Cancer Trialist’s Collaborative Group, the benefit from adjuvant BPs is lower in more recent clinical trials, especially in patients receiving contemporary chemotherapy. The balance between benefits and risks of adjuvant BPs should be considered in individual patients.

## 5. Oral Presentation

**Rates of Surgery and Adjuvant Chemotherapy Use in Stage IB-IIIA Non-Small Cell Lung Cancer Patients: An Ontario Population-Based Study**
Yuchen Li ^1^, Gregory Pond ^2^, Yaron Shargall ^3,4^, Housne Begum ^4,5^ and Rosalyn Juergens ^1^

1. Department of Oncology, Juravinski Cancer Centre, Hamilton Health Sciences, Hamilton, Ontario
2. Ontario Institute for Cancer Research, Ontario
3. Division of Thoracic Surgery, St. Joseph’s Healthcare System, Hamilton, ON
4. The Division of Thoracic Surgery, McMaster University, Hamilton, ON
5. Health Services Management Department, Toronto Metropolitan University, Toronto, ON

**Objective**

To identify the rates of adherence to guideline-recommended surgery and adjuvant chemotherapy treatment for early-stage (IB to IIIA) non-small cell lung cancer (NSCLC) patients in Ontario.

**Methods**

A retrospective population-based study using linked administrative data through ICES was completed that included all adult patients (age of 18 years or older) that were diagnosed with stage IB to IIIA (AJCC 7th edition) NSCLC from 2010 to 2020 in Ontario. The rates of surgery and chemotherapy completion were calculated using available OHIP (Ontario Health Insurance Plan) billing codes. A logistic regression was also completed to assess any predictors for adherence to the guideline-recommended treatments.

**Results**

A total of 24,237 eligible patients were included. By cancer staging, there were 6495 (26.8%) stage IB, 7156 (29.5%) stage II, and 10,586 (43.7%) stage IIIA NSCLC patients. Within 180 days of diagnosis, surgery was completed for 9929 (41.0%) patients; by cancer staging, the numbers were 4090/6495 (63.0%), 3719/7156 (52.0%), and 2120/10,586 (20.0%) for IB, II, and IIIA, respectively. The median time from diagnosis to surgery was 49 days (IQR 23–77 days). Amongst patients who completed surgery within 180 days, 3344/9929 (33.7%) commenced adjuvant chemotherapy within 180 days. The median time from diagnosis to first chemotherapy administration was 71 days (IQR 44–106 days).

**Conclusions**

In our population-based study, less than half of the patients with early-stage (IB-IIIA) NSCLC underwent surgical resection and chemotherapy. These low rates are concerning and are likely leading to sub-optimal outcomes. Furthermore, in the context of increasing the number of neoadjuvant and adjuvant clinical trials involving early-stage NSCLC patients, the real-world outcomes may be drastically different from the trial outcomes if there is poor adherence to guidelines in the real world. Our data suggest a need for quality improvement strategies to identify and improve treatment adherence for patients with early-stage NSCLC.

**Award Recipient**

## 6. Oral Presentation

**The Impact of COVID-19 On The Wellness and Resilience of The Canadian Medical Oncology Workforce: A Canadian Association of Medical Oncologists Survey**
Lauren Jones ^1^, Bruce Colwell ^2^, Desiree Hao ^3^, Stephen Welch ^4^, Alexi Campbell ^5^ and Sharlene Gill ^1^

1. Medical Oncology, BC Cancer, University of British Columbia, Vancouver, BC
2. Medical Oncology, Dalhousie University, Halifax, NS
3. Medical Oncology, Tom Baker Cancer Centre, Calgary, AB
4. Medical Oncology, London Regional Cancer Program, London, ON
5. Canadian Association of Medical Oncologists, Ottawa, ON

**Background**

The COVID-19 (C19) pandemic has presented professional and personal challenges. The Canadian Association of Medical Oncologists (CAMO) has been examining the effects of C19 on the workforce to understand the impact of the pandemic on the medical oncology (MO) community. This survey examines how C19 has impacted the workforce with a focus on wellness and resilience; it also assesses the impact that C19 may have on the MO workforce capacity going forward.

**Methods**

An English-language, multiple-choice survey was distributed by email to MO members who were identified through CAMO and the Royal College of Physicians and Surgeons directory in March 2022.

**Results**

The response rate was 32% (*n* = 151/477). The respondents were 59% female, where 88% worked in comprehensive cancer centers and 64% had been in practice for > 10 years. Physical (60%) and mental (60%) wellness were reported as the biggest personal challenges. Of the respondents, 47% were dissatisfied or very dissatisfied with their current work–life balance, and 83% indicated that their workload had increased since the beginning of C19. Of the respondents, 56% were considering retiring or reducing total working hours or full-time equivalent (FTE) in the next 5 years, and 35% had considered leaving the MO community entirely. Career length > 10 years and age > 40 years were associated with considering leaving MO (*p* = 0.01 and *p* = 0.03, respectively). Career length > 10 years was associated with the consideration of reducing total working hours or current FTE within the next 5 years (*p* = 0.045) (Table 1).

**Conclusion** **s**

This survey corresponds with the transition of the C19 pandemic towards becoming endemic. There are concerns that correspond to physician wellness, workload escalation, and job dissatisfaction. One-third of respondents were considering leaving MO practice, which was associated with > 10 years of practice, suggesting the potential loss of the senior, experienced workforce. With the escalating demand for MO services due to rising cancer prevalence and treatment complexity, the proactive implementation of wellness, retention, and workload modification strategies are needed to ensure the stability of the Canadian MO workforce.

**Table 1:** Univariate analysis of categorical variables among respondents that are considering leaving medical oncology.

	Considering Leaving Medical Oncology		Considering Reducing Hours/FTE	
Sex				
Female	53%	*p* = 0.23	59%	*p* = 0.69
Male	45%		40%	
Age				
<40	12%	*p* = 0.03	20%	*p* = 0.43
>40	88%		80%	
Practice setting				
Comprehensive cancer center	94%	*p* = 0.08	89%	*p* = 0.58
Other	6%		11%	
Years in practice				
<10	23%	*p* = 0.01	30%	*p* = 0.045
>10	77%		70%	
Feel valued by institution				
Yes	27%	*p* = 0.98	24%	*p* = 0.36
No	73%		76%	
Feel valued by public				
Yes	38%	*p* = 0.70	45%	*p* = 0.26
No	62%		55%	

## 7. Oral Presentation

**A Retrospective Review of Primary Prophylaxis with Granulocyte Colony-Stimulating Factor (G-Csf) for Patients With Genitourinary Malignancies Receiving Chemotherapy During the COVID-19 Pandemic and Implications for The Future**
Nely Diaz-Mejia, Carlos Stecca, Di Maria Jiang, Nazanin Fallah-Rad, Philippe Bedard, Kumar Vikaash, Osama Abdeljalil, Amer Zahralliyali, Husam Alqaisi, Esmail Al-ezz, Vivian Choy, Parmvir Banwait, Eshetu G. Atenafu and Srikala S. Sridhar
Division of Medical Oncology and Hematology, Princess Margaret Cancer Centre, Toronto, ON

**Background**

To mitigate the risks of chemotherapy-associated neutropenia, during the COVID-19 pandemic, all genitourinary (GU) cancer patients that were treated with chemotherapy at the Princess Margaret Cancer Centre (PMCC) were offered primary prophylaxis with GCSF. We hypothesized that these reduced the rates of febrile neutropenia, hospitalizations, and healthcare costs and improved the overall outcomes compared with GU cancer patients treated with chemotherapy without GCSF in the 2 years prior to the pandemic.

**Methods**

We performed a retrospective review of GU cancer patients receiving curative or palliative intent chemotherapy and with or without primary GCSF prophylaxis between Jan 2018 and June 2022.

**Results**

Overall, 248 patients with prostate cancer (44%), urothelial cancers (33%), germ cell (21%), and rare GU cancers (4%) were identified. The median age was 70 years (range 19–91 years), 92% were male, and 65% were ECOG 0/1. The treatment intent was neoadjuvant (13%), adjuvant (20%), or palliative (67%). The main regimens used were docetaxel, cabazitaxel, carboplatin, cisplatin/etoposide, gemcitabine/cisplatin, and BEP. Median follow-up was 10.5 months (0.23–52.3 months). A total of 206/248 patients received primary GCSF prophylaxis.

During chemotherapy, the median white blood cell levels were higher in the GCSF group compared with the non-GCSF group (14.1 × 10 × 9/L vs. 2.90 × 10 × 9/L, *p* < 0.0001); neutropenia rates were markedly lower (2% vs. 93%, *p* = < 0.0001). Hospital admission rates were significantly lower in G-CSF users compared with non-users (19% vs. 69%, *p* < 0.0001). A symptomatic disease progression of 13% was the leading cause of admission in the G-CSF group. Infectious causes such as UTI, pneumonia, COVID-19, and sepsis were seen in 12% of the G-CSF group compared with 31% in the non-users. G-CSF was well tolerated, with just 0.97% discontinuing G-CSF.

**Conclusions**

During the COVID-19 pandemic, primary prophylactic G-CSF use in GU cancer patients undergoing chemotherapy significantly lowered the rates of both febrile neutropenia and hospitalizations; this could be a cost-effective strategy in this patient population that warrants further study.

## 8. Oral Presentation

**Real-World Evaluation of Bone-Targeted Agents and First Bone Radiation Incidence in Prostate Cancer Decedents: A Provincial-Wide Population Study**
William J Phillips ^1^, Jennifer Leigh ^1^, Alborz Jooya ^2^, Anan Eddeen ^3^, Christina Milani ^3^, Scott Morgan ^2^, Rob Macrae ^2^, Peter Tanuseputro ^3^, Michael Ong ^4^ and Jean-Marc Bourque ^5^

1. University of Ottawa Department of Medicine, Ottawa, ON
2. University of Ottawa, Department of Radiation Oncology, Ottawa, ON
3. The Ottawa Hospital Research Institute, Ottawa ON
4. The Ottawa Hospital Centre, Ottawa, ON
5. Integrate Cancer Centre CHUM, Montreal Quebec

**Objective**

To evaluate the use of bone-targeted agents (BTAs) and its relationship with palliative bone radiation as a measure of skeletal-related events (SREs) in patients with metastatic castration-resistant prostate cancer (mCRPC).

**Methods**

Provincial-wide administrative databases identified patients with prostate cancer (2007–2018, *n* = 98,646) who received continuous androgen deprivation therapy (*n* = 29,453), died of prostate cancer (2013–2018, *n* = 3864), and received life-prolonging therapy for mCRPC (LPT: abiraterone, enzalutamide, docetaxel, cabazitaxel and radium-223, *n* = 1850). The demographic-, clinical-, and cancer-related variables were collected using a 3-year observation window from the date of death.

**Results**

Of the 1850 patients who met the inclusion criteria, 1066 (58%) received BTAs and 1137 (62%) received palliative bone radiation. More patients received denosumab (*n* = 825, 77%) than zoledronic acid (*n* = 241, 23%). A total of 289 patients (25.4%) started BTA prior to first bone radiation, while 848 patients (74.6%) either did not receive BTAs (*n* = 447, 53%) or started BTAs after first bone radiation (*n* = 401, 47%). A total of 376 patients received BTAs and never received bone radiation. BTAs were started within 25–36 months (*n* = 294, 28%), 13–24 months (*n* = 334, 31%), or 12 months (*n* = 438, 41%) relative to death. First bone radiation occurred within 25–36 months (*n* = 188, 16%), 13–24 months (*n* = 367, 32%), or 12 months (*n* = 582, 51%) relative to death. Factors associated with the receipt of BTAs included palliative bone radiation (*p* = 0.008), radiation to non-bone (*p* = 0.035), elevation in ALP (*p* < 0.001), prior prostatectomy (*p* = 0.008), younger age (*p* < 0.001), medical oncology involvement (*p* < 0.001), and palliative care involvement (*p* = 0.0045).

**Conclusions**

Patients receiving contemporary prostate cancer treatments still suffer from a significant burden of skeletal-related events, and the majority only started BTA treatment after first having an SRE. Our results highlight an opportunity to improve outcomes by emphasizing the early introduction of BTAs in mCRPC patients who are being started on LPT.

## 9. Oral Presentation

**Anemia is Inversely Associated with Patient Survival in Melanoma Patients Treated With Immunotherapy**
Hyejee Ohm ^1,2^, Derek Tilley ^3^, Hatim Karachiwala ^4,5^ and John Walker ^2,6^

1. Department of Internal Medicine, Edmonton, AB
2. University of Alberta, Edmonton, AB
3. Alberta Health Services, Calgary, AB
4. University of Calgary, Calgary, AB
5. Tom Baker Cancer Centre, Calgary, AB
6. Cross Cancer Institute, Edmonton, AB

**Objective**

Emerging evidence indicates that anemia may confer an immunosuppressive state, including for patients with cancer. We hypothesized that the presence of anemia may negatively impact the efficacy of immunotherapy. We tested for associations between peripheral blood cell counts (hemoglobin, leukocyte, neutrophil, lymphocyte, and platelet counts) and the response to immunotherapy as measured by overall survival (OS) and objective response rate (ORR).

**Methods**

A retrospective cohort study was conducted on Albertan patients > 18 years old with unresectable or metastatic melanoma diagnosed between January 2008 and December 2017 and who were treated with at least one dose of immunotherapy. The data were extracted from the Alberta Cancer Registry. Retrospective chart reviews were completed for the laboratory data and radiological findings. Tests for associations between peripheral blood counts (hemoglobin, leukocyte, neutrophil, lymphocyte, and platelet counts) and OS/ORR were performed. The analyses were adjusted for confounders, including age, sex, toxicity, Charlson Comorbidity Index, transfusion history, tumor location, and treatment regimen.

**Results**

Among 377 patients, 36.6% received ipilimumab, 45.3% received PD-1 monotherapy, and 17.7% received CTLA4/PD-1 combination therapy. Anemia (HR 1.77, 95% CI [1.19–2.52], *p* = 0.005) was independently associated with a reduced OS, and anemia was associated with a significantly lower ORR (41.3% vs. 27.6%, *p* = 0.007). Unsurprisingly, abnormal leukocyte counts also correlated with reduced efficacy, but by comparison, no statistically significant associations were found between low platelet counts and OS or ORR.

**Conclusions**

In our analysis, OS was proportionally diminished with the degree of anemia, but thrombocytopenia was not; this suggests that our observation has immunological significance. Anemia and lymphopenia, but not abnormal platelet counts, were associated with reduced OS among metastatic melanoma patients. Only anemia was associated with a reduced ORR. Our analysis is limited by its retrospective nature but suggests that anemia may negatively impact immunotherapy treatment outcomes. More study is required to delineate possible immunologic mechanisms to explain our findings.

## 10. Oral Presentation

**Androgen Receptor is Expressed in the Majority of Breast Cancer Brain Metastases and is Subtype-Dependent**
Kevin Yijun Fan ^1^, Rania Chehade ^2^, Maleeha Qazi ^1^, Veronika Moravan ^3^, Sharon Nofech-Mozes ^4^ and Katarzyna Jerzak ^5^

1. Faculty of Medicine, University of Toronto, Toronto, Ontario
2. Department of Medical Oncology, Faculty of Medicine, University of Toronto, Toronto, Ontario
3. VM stats, Toronto, Ontario
4. Department of Laboratory Medicine and Pathobiology, University of Toronto, Toronto, Ontario
5. Sunnybrook Odette Cancer Centre, University of Toronto, Toronto, Ontario

**Objective**

Given the availability of central nervous system (CNS)-penetrant systemic therapies that target the androgen receptor (AR), we evaluated the expression of the AR “target” in breast cancer brain metastases (BrMs).

**Methods**

An established, retrospective cohort of 57 patients with metastatic breast cancer who underwent surgery for BrMs at the Sunnybrook Odette Cancer Centre (SOCC) between 1999 and 2013 was studied. AR expression in BrM samples was assessed in triplicate using immunohistochemistry (IHC). AR-positive status was defined as nuclear AR expression ≥ 10% in tumor-infiltrating cells as a percentage of tumor area using the SP107 antibody.

**Results**

The median age of patients was 52 years (range 32–85 years). A total of 17 (30%) patients had hormone receptor-positive (HR+)/HER2-negative, 28 (49%) had HER2+, and 12 (21%) had triple-negative breast cancer (TNBC) BrM. Out of the patients, 61.4% (*n* = 35) had a single BrM, and the median size of BrM was 3 cm (ranging from 0.3 cm to 6.2 cm). The median expression of AR was 20% (CI 1.6–38.3%), and 32 out of 57 (56%) BrMs were AR-positive based on a cut-point of ≥ 10%. A significantly smaller proportion of patients with TNBC had AR+ BrM (*n* = 2/12, 17%) compared with patients with HR+/HER2-negative (*n* = 9/17, 53%) or HER2+ (*n* = 21/28, 75%) disease (*p* = 0.04). Patients with AR-positive versus AR-negative BrMs had similar overall survival (12.5 vs. 7.9 months, *p* = 0.6), brain-specific progression-free survival (8.0 vs. 5.1 months, *p* = 0.95), and time from breast cancer diagnosis to BrM diagnosis (51 vs. 29 months, *p* = 0.16). In a subset of 10 patients for whom matched primary breast tumor tissue was available, the AR status was concordant in 7 (70%) cases.

**Conclusions**

AR is expressed in the majority of breast cancer BrMs and represents a promising therapeutic target.

## 11. Oral Presentation

**Informative Tools to Optimize Neoadjuvant Therapy in Er-Positive, Her2-Negative Breast Cancers**
Lidiya Luzhna, Stephen Chia, Mehrnoosh Pauls and Nathalie Levasseur
BC Cancer Vancouver, Vancouver, BC

**Background**

Neoadjuvant therapy (NAT) in HR+/HER2- tumors is often disputable. While the role of Oncotype DX has been well established in adjuvant settings, its clinical utility and potential implementation for the prediction of NAT benefit requires further study.

**Purpose**

This ongoing study aims to report the feasibility of Oncotype DX testing on core biopsy specimens prior to NAT in patients with operable HR+/HER2- breast cancer. The effect of the recurrence score (RS) on systemic treatment recommendations and its correlation to dynamic markers of proliferation and NAT response will be evaluated.

**Methods**

Participants with clinical T2–T4 and/or node-positive HR+/HER2- breast cancer had Oncotype DX testing conducted prior to NAT. The clinico-pathological information, treatment regimens, and clinical, radiologic, and pathological responses were recorded.

**Results**

Of the 48 patients with HR+/HER2- breast cancer that were referred to BC Cancer Vancouver for consideration of NAT between September 2021 and January 2023, 26 were eligible and enrolled in the study. The success rate of Oncotype DX testing on core biopsy samples was 96%. The mean turnaround time from patient consent to RS report was 19 calendar days. Approximately 32% of tumors had an RS equal or higher than 26, while 4% of tumors had a score less than 10. Nearly 25% of neoadjuvant treatment recommendations were changed based on the RS. Overall, 33% of patients received neoadjuvant chemotherapy, 54% received neoadjuvant endocrine treatment, and 12.5% proceeded with upfront surgery.

**Conclusions**

The success rate of Oncotype DX testing on core biopsy samples was excellent. There were multiple factors that influenced trial enrollment, highlighting the low uptake and equipoise of NAT use in HR+/HER2- tumors. The Oncotype DX testing resulted in a change in the systemic treatment plan in 25% of patients. Analyses are ongoing to correlate the RS to NAT response, dynamic Ki-67 changes, and breast MRI changes.

## 12. Poster Presentation

**Test Performance and Clinical Validity of Circulating Tumor Dna (Ctdna) in Predicting Relapse in Solid Tumors Treated with Curative Intent Therapy**
Abhenil Mittal ^1^, Consolacion Molto Valiente ^1^, Faris Tamimi ^1^, Massimo Di Iorio ^2^, Laith Al-Showbaki ^1^, David Cescon ^3^ and Eitan Amir ^3^

1. Clinical Fellow, Division of Medical Oncology and Hematology, Princess Margaret Cancer Center, Toronto, ON
2. Medical Oncology Resident, Division of Medical Oncology and Hematology, Princess Margaret Cancer Center, Toronto, ON
3. Division of Medical Oncology and Hematology, Princess Margaret Cancer Center, Toronto, ON

**Background**

The presence of circulating tumor DNA (ctDNA) is prognostic in solid tumors that are treated with curative intent. Studies have evaluated ctDNA at specific ‘landmark’ timepoints or over numerous ‘surveillance’ timepoints. However, variable results have led to uncertainty about the clinical validity of this tool.

**Methods**

A search of MEDLINE (host: PubMed) identified studies evaluating ctDNA monitoring after curative intent therapy in solid tumors. The odds ratios (OR) for clinical and/or radiologic recurrence at both landmark and surveillance time points for each study were calculated and pooled in a meta-analysis using the Peto method. The pooled sensitivity and specificity as weighted by an individual study inverse variance method were estimated; meta-regression utilizing linear regression weighted by inverse variance was performed to explore the associations between patient and tumor characteristics and the OR for disease recurrence.

**Results**

Of the 39 studies identified; 30 (1924 patients) and 24 studies (1516 patients) reported on landmark and surveillance time points, respectively. The pooled OR for recurrence at the landmark was 15.47 (95% CI 11.84–20.22); at the surveillance point, it was 31.0 (95% CI 23.9–40.2). The pooled sensitivity for ctDNA at the landmark and surveillance analyses were 58.3% and 82.2%, respectively. The corresponding specificities were 92% and 94.1%. There was lower prognostic accuracy with the use of tumor agnostic panels. Adjuvant chemotherapy negatively impacted specificity in the landmark setting. Longer time to landmark analysis, higher number of surveillance blood draws, and a history of smoking were associated with higher prognostic accuracy.

**Conclusions**

Although ctDNA at both landmark and surveillance time points shows high prognostic accuracy for relapse in patients with solid tumors treated with curative intent, it has low sensitivity, borderline high specificity, and thus modest discriminatory accuracy, especially for landmark analyses. Adequately designed clinical trials with appropriate testing strategies and assay parameters are required to demonstrate clinical utility.

**Award Recipient**

## 13. Poster Presentation

**Immune Checkpoint Inhibitors in the Treatment of Small Cell Lung Cancer: A Systematic Review and Meta-Analysis**
Nupur Krishnan ^1^, Patsy Wai Pin Lee ^2^, Gabriel Boldt ^3^, Suganija Lakkunarajah ^4^, Phillip Blanchette ^5^ and Jacques Raphael ^5^

1. Faculty of Health Sciences, McMaster University, Hamilton, Ontario
2. Department of Internal Medicine, Schulich School of Medicine and Dentistry, Western University, London, Ontario,
3. Department of Radiation Oncology, London Health Sciences Centre, London, Ontario
4. Department of Medicine, Schulich School of Medicine & Dentistry, Western University, London, Ontario
5. Division of Medical Oncology, Department of Oncology, London Regional Cancer Centre, London Health Sciences Centre and Western University, London, Ontario

We conducted a systematic review and meta-analysis to assess the role of immune checkpoint inhibitors (ICIs) in the treatment of extended-stage small cell lung cancer (ES-SCLC).

The MEDLINE (PubMed), EMBASE, and Cochrane Library databases were queried between January 2010 and March 2022, and conference proceedings between 2018 and 2022 were searched for randomized clinical trials assessing ICIs (combined with chemotherapy or as single agents), compared with chemotherapy, in patients with ES-SCLC. The primary endpoints were overall survival (OS) and progression-free survival (PFS). Secondary endpoints included objective response rate (ORR) and grade 3 or higher adverse events (AEs). Pooled hazard ratios (HRs) for OS and PFS were meta-analyzed using the generic inverse variance method, and random-effect models were used to compute pooled estimates. Subgroup analyses compared the survival by line of therapy, sex, age, and ECOG status.

A total of 5325 patients from 10 trials were included. Compared with chemotherapy, ICI-based treatment decreased the risk of death by 19% (HR 0.81, 95% confidence interval (CI) 0.76–0.86). The OS benefit was observed regardless of age, sex, or ECOG status, but was only observed in patients treated in the first-line setting. Similarly, ICI-based therapy decreased the risk of disease progression by 20% (HR 0.80, 95% CI 0.68–0.94), and the PFS benefit was restricted to first-line treatment with a detrimental effect occurring in the second-line setting. The ORR was also improved with ICIs (odds ratio (OR) 0.80, 95% CI 0.65–0.97) with an increase in grade 3 or higher diarrhea (OR 3.63, 95% CI 1.46–9.02).

In conclusion, ICIs conferred efficacy benefits (OS, PFS, and ORR) and an acceptable safety profile in the treatment of patients with ES-SCLC in the first-line setting; they should not be used in the second-line setting as single agents. Valid biomarkers that predict the long-term benefits are needed to further improve outcomes.

## 14. Poster Presentation

**Assessment of Tumor-Infiltrating Lymphocytes as A Prognostic Factor In Patients with Advanced Melanoma Treated with Immunotherapy**
Zahra Taboun ^1^, Jasna Deluce ^1,2^, Adi Kartolo ^3^, Wilma Hopman ^4^, Tara Baetz ^5^ and John Lenehan ^1,2^

1. Schulich School of Medicine and Dentistry, Western University, London, ON
2. Department of Oncology, London Health Sciences Centre, London, ON
3. Department of Oncology, Juravinski Cancer Centre, McMaster University, Hamilton, ON
4. Public Health Sciences, Queen’s University, Kingston, ON
5. Department of Oncology, Cancer Centre of Southeastern Ontario, Kingston, ON

**Objective**

To determine whether tumor-infiltrating lymphocytes (TILs) impact prognosis in patients treated with immunotherapy for advanced melanoma.

**Methods**

This retrospective study involved 204 patients with metastatic melanoma who received single-agent, palliative-intent PD-1 inhibitors between January 2012 and December 2021 at the Cancer Centre of Southeastern Ontario (CCSEO) and London Regional Cancer Program (LRCP). TILs were assessed in the pathology specimen prior to immunotherapy initiation. TIL status was defined as ‘brisk’, ‘non-brisk/absence’, or ‘unknown’. The Primary study outcome was overall survival (OS).

**Results**

A total of 28 (14%), 88 (43%), and 88 (43%) patients had ‘brisk’, ‘non-brisk/absence’, and ‘unknown’ TIL statuses, respectively. Patients with cutaneous melanoma were more likely to have ‘brisk’ TIL status when compared with non-cutaneous melanoma (13% vs. 1%, *p* = 0.022). The estimated median OS with 95% confidence interval (CI) of the TIL brisk, TIL Non-brisk/absent, and TIL unknown were 30.5 (95% CI 12.2–48.8) months, 16.4 (95% CI 10.7–22.1) months, and 15.9 (95% CI 9.2–22.6) months, respectively, albeit this result was statistically non-significant (*p* = 0.57). A multivariate analysis showed no OS improvement when ‘brisk’ was compared with ‘non-brisk/absence’ TIL statuses (HR 0.75 95% CI 0.42–1.32); rather, ECOG ≥2 (HR 2.03, 95% CI 1.35–3.05) and elevated LDH levels (HR 2.06, 95% CI 1.43–2.95) were independent prognostic factors for a worse OS.

**Conclusions**

Our study demonstrated a non-significant improved OS trend favoring ‘brisk’ over ‘non-brisk/absence’ or ‘unknown’ TIL statuses in advanced melanoma patients on immunotherapy. However, our study was limited by its small sample size and relatively large ‘unknown’ TIL group. If validated in future larger, multi-center studies, TILs may become part of standard of care pathology reporting, given its inexpensive and benefit of not requiring an additional invasive procedure.

**Award Recipient**

## 15. Poster Presentation

**An Examination of the Determinants and Outcomes of Acute Immune Checkpoint Inhibitor (Ici) Pneumonitis in Patients with Locally Advanced Non-Small Cell Lung Cancer (La-Nsclc) Receiving Durvalumab Consolidation Following Chemoradiation: A Retrospective, Population-Based Multicenter Study**
Chloe Lim ^1^, Sunita Ghosh ^2^, Daniel Meyers ^1^, Igor Stukalin ^1^, Marc Kerba ^3^, Desiree Hao ^3^, Aliya Pabani ^3^

1. Internal Medicine Residency Program, University of Calgary, Calgary, AB
2. Cancer Care Alberta, University of Alberta, AB
3. Tom Baker Cancer Center, Department of Oncology, University of Calgary, AB

**Background**

Consolidation durvalumab following chemoradiotherapy has improved survival outcomes in patients with LA-NSCLC. However, ICI pneumonitis is a serious complication that can have life-threatening outcomes, necessitating ICI discontinuation. Understanding the factors that drive the risk of pneumonitis can inform patient selection and treatment monitoring. The objective of this study was to characterize the risk factors and outcomes of ICI pneumonitis in NSCLC patients treated with consolidation durvalumab therapy in real-world practices.

**Methods**

Using the Alberta Immunotherapy Database, we retrospectively evaluated all NSCLC patients who received durvalumab in Alberta, Canada, from Jan/18 to Dec/21. Pneumonitis cases were identified based on radiographic changes and oncologists’ clinical assessments. We examined the incidence and predictive values of severe pneumonitis, with the secondary outcomes of overall survival (OS) and time-to-treatment failure (TTF). Exploratory multivariate analyses were performed to identify the predictive values for developing severe pneumonitis.

**Results**

Out of 189 patients, most were ECOG 0–1 (91%) and partial response from chemoradiation (85%) prior to durvalumab. The results showed that 49% of patients received a full year of therapy (*n* = 93). Median TTF = 11.2 months; median OS = 19.7 months with 1-year OS = 64% (*n* = 121). Median treatment duration = 62 days with a median follow-up duration of 613 days. Durvalumab was discontinued in 23% of patients due to any toxicity. It was found that 26% (*n* = 49) of patients developed any grade of pneumonitis, and 9% (*n* = 17) of patients had ≥ grade 3 pneumonitis. With respect to deaths, 13% (*n* = 9) were attributed to pneumonitis. Corticosteroids were administered to 86% of the patients with pneumonitis (*n* = 42). Male sex and a pre-existing autoimmune condition were associated with ≥ grade 3 pneumonitis.

**Conclusions**

We report a risk of pneumonitis that is comparable to prior retrospective studies and the PACIFIC study, but a higher incidence of corticosteroid use. This is the first Canadian real-world study to date that has explored clinical factors associated with ICI pneumonitis post-durvalumab therapy in NSCLC, which may help guide future patient selection for the safe completion of consolidation immunotherapy.

## 16. Poster Presentation

**Real-World Sequence of Peptide Receptor Radionucleotide Therapy (Prrt) and Chemotherapy in Patients with Metastatic Pancreatic Neuroendocrine Tumors (Pnets)**
William J Phillips ^1^,Elena Tsvetkova ^2^, Macyn Leung ^3^, Gautham Nair ^4^, Stephen Welch ^2^, David Laidley ^2^, Tim Asmis ^5^, Michael Vickers ^5^, Morgan Black ^2^, Robin Sachdeva ^2^, Horia Marginean ^3^, Rachel Goodwin ^5^

1. Department of Medicine University of Ottawa, Ottawa, ON
2. London Regional Cancer Program (LRCP), London, ON
3. The Ottawa Hospital Research Institute, Ottawa, ON
4. Faculty of Medicine Western University, London, ON
5. The Ottawa Hospital Cancer Centre, Ottawa, ON

**Introduction**

To characterize the sequence of PRRT and chemotherapy at two Canadian pNET treatment centers, as distinguished by the availability of PRRT.

**Methods**

This is a multicenter retrospective cohort study involving the Ottawa Hospital Cancer Centre (TOHCC; PRRT non-treatment center (PRRT-NTC)) and London Regional Cancer Centre (LRCC; PRRT treatment center (PRRT-TC). Patients with histologically confirmed pNETs between January 2010 and June 2021 over the age of 18 years (*n* = 226) with metastatic disease (*n* = 177) were included. Demographic, clinical, and cancer treatments were collected. Descriptive statistics were used to evaluate the sequence of systemic therapy.

**Results**

A total of 177 patients with metastatic pNET were identified. The most common locations of metastasis were in livers (*n* = 135, 76%), lymph nodes (*n* = 65, 37%), and bones (*n* = 37, 21%). Systemic therapies included octreotide (*n* = 59, 40%), lanreotide (*n* = 47, 32%), targeted therapy (*n* = 40, 27%), chemotherapy (*n* = 48, 33%), and PRRT (*n* = 47, 32%). Temozolomide-containing chemotherapy was used in 22 (46%) patients.

There were 83 (47%) patients that were treated at the PRRT-TC. Of those, 44 (53%) patients received PRRT and chemotherapy, 11 (25%) of which received chemotherapy prior to PRRT. Likewise, 33 (35%) patients received PRRT and chemotherapy at the PRRT-NTC, 1 (3%) of which received chemotherapy prior to PRRT. There were seven (16%) patients who received temozolomide prior to PRRT at the PRRT-TC compared with zero (0%) patients at the PRRT-NTC.

**Discussion**

Early data from United States centers may suggest an association with temozolomide prior to PRRT and the development of myelodysplasia and leukemia. In this large real-world sample, sequencing of chemotherapy and PRRT showed center-to-center variation, with metastatic pNET patients treated at a PRRT-TC being more likely to receive chemotherapy, including temozolomide, prior to PRRT. This may be explained in part by referral/practice patterns and PRRT availability. These findings suggest an opportunity to evaluate and optimize the sequence of systemic therapy with the increasing availability of PRRT in Canada.

## 17. Poster Presentation

**Incidence of Radiotherapy for Brain Metastases among Breast Cancer Patients in Ontario: A Population-Based Study**
Rania Chehade ^1^, Xin Ye Wang ^2^, Michael N. Rosen ^3^, Arjun Sahgal ^4^, Sunit Das ^2,5^, Refik Saskin ^6^, Bo Zhang ^6^, Hany Soliman ^4^, Kelvin K.W. Chan ^1,2^ and Katarzyna J. Jerzak ^1,2^

1. Division of Medical Oncology, Sunnybrook Odette Cancer Centre, University of Toronto, Toronto, Ontario, Canada
2. Institute of Medical Sciences, Faculty of Medicine, University of Toronto, Toronto, Canada
3. Faculty of Medicine, University of Ottawa, Ottawa, Canada
4. Department of Radiation Oncology, Sunnybrook Odette Cancer Centre, University of Toronto, Toronto, Ontario, Canada
5. Division of Neurosurgery, St. Michael’s Hospital, University of Toronto, Toronto, Canada
6. Institute of Health Policy, Management and Evaluation, University of Toronto, Toronto, ON, Canada; Institute of Clinical Evaluative Sciences, Sunnybrook Health Sciences Centre, Sunnybrook Research Institute, Toronto, ON, Canada

**Objective**

To evaluate the cumulative incidence of radiotherapy for brain metastases (BrMs) among patients diagnosed with breast cancer (BC) in Ontario.

**Methods**

We conducted an Ontario-wide, retrospective, observational cohort study using the ICES database to assess treatment patterns and outcomes of patients with BC who received radiotherapy for BrMs between January 2009 and December 2018.

The primary endpoint was the cumulative incidence of radiotherapy for BrMs accounting for the competing risk of death. The data were censored if patients were alive on the same therapy at the last available follow-up with the last cut-off date being 31 March 2019. Kaplan–Meier analyses were performed for the time-to-event endpoints and were compared using the log-rank test. The cumulative incidence of radiotherapy for BrMs from the diagnosis of BC was calculated using the cumulative incidence function while accounting for the competing risk of death using a competing risk analysis. Multivariable regression models were used to account for confounding variables.

**Results**

A total of 88,111 patients with BC were identified; of these patients, 85.2% had a stage I–III disease and 4.6% had a stage IV disease at diagnosis. In the overall population, 2.4% (*n* = 2120) received radiotherapy for BrMs; this proportion was highest among patients with a de novo stage IV disease (14%, *n* = 549). Out of the patients with BrMs, 82% (*n* = 1738) were treated using whole brain radiotherapy (WBRT) while 18% (*n* = 382) were treated using stereotactic radiation (SRS). Patients treated using SRS had a longer median OS compared with those treated using WBRT (9.3 months vs. 4.6 months, *p* < *0*.0001) with a lower 30-day mortality (5.2% vs. 15.5%, *p* < 0.001) after adjustment for confounding variables.

**Conclusions**

Approximately one in seven patients with metastatic BC will require radiotherapy for BrMs. Given that women treated for BC BrMs using SRS had better outcomes, the use of SRS should be encouraged when clinically indicated.

## 18. Poster Presentation

**Multidisciplinary Rectal Cancer Rounds—Characterization of Delays to Oncological Consultation and Treatment among Patients scheduled For Clinical Discussions; Quality Improvement Analysis**
Joanna Mycek ^1^, Mark Freeman ^2^, Richard Lee-Ying ^2^ and Hyea Min Yoon ^3^

1. Department of Medicine, University of Calgary, Alberta, Canada
2. Department of Oncology, Tom Baker Cancer Centre, Calgary, Alberta, Canada
3. Department of Surgery, University of Calgary, Alberta, Canada

**Objective**

Since September 2020, the Tom Baker Cancer Centre (TBCC) has embedded weekly multidisciplinary rounds reviews of all non-metastatic rectal cancer cases to determine optimal management before formal consultations. However, this extra step could lead to delays in management.

**Methods**

We reviewed all non-metastatic rectal cancer referrals from January 2019 to December 2021 and compared the time from clinical diagnosis to first TBCC consultation (either a medical or radiation oncologist) and time to first rectal cancer treatment with those reviewed in rounds and those that were not.

**Results**

During the study period, 203 eligible patients were identified, with a median age of 63 years (IQR 53–70); the patients were predominantly male (62.6%). The majority, i.e., 126 (62.0%) patients, had a stage III disease. In total, 126 (62.1%) patients received neoadjuvant therapy with a predominance of long-course chemoradiation (89, 43.8%). Only 11 (5.4%) patients received total neoadjuvant therapy. Surgical resection occurred in 168 (82.8%) patients, with low anterior resection being the most common procedure (124, 61.1%). Adjuvant treatment was administered in 115 (56.7%) patients, which was principally conducted using chemotherapy alone (98, 48.3%). A total of 97 (47.8%) patients were reviewed in rounds, whereas 106 (52.2%) were not. Following multidisciplinary review, 25 (12.3%) patients were downstaged and 9 (4.4%) were upstaged. Among the patients reviewed and among those that were not, the median time from diagnosis to TBCC consultation was 41 days (IQR 27.5–74.0) and 57 days (IQR 28.0–90.5), respectively (*p* = 0.926), and the median time from diagnosis to first treatment was 53 (IQR 40.0–68.5) and 49 days (IQR 37.5–63.8), respectively (*p* = 0.284).

**Discussion**

This convenience sample demonstrates no statistically significant delays between diagnosis and first oncological consultation or treatment among patients that were scheduled for mandatory multidisciplinary rounds discussions. These findings provide reassurance that adding steps to improve the quality of rectal cancer management does not have a worrisome impact on time to consultation or treatment.

## 19. Poster Presentation

**Multisystem Immune-Related Adverse Events from Dual Agent Immunotherapy Use**
**Yuchen Li ^1^, Gregory Pond ^2^ and Elaine McWhirter ^1^**

1. Department of Oncology, Juravinski Cancer Centre, Hamilton, Ontario
2. Ontario Institute for Cancer Research, Ontario

**Objective**

To assess the rate of multisystem immune-related adverse events (irAEs) for patients who received ipilimumab and nivolumab for cancer treatment.

**Method**

A retrospective cohort review was completed that included all cancer patients seen at Juravinski Cancer Centre who received at least one dose of ipilimumab and nivolumab from 1 January 2018 to 31 May 2022. The patient characteristics, cancer types, and number and types of irAEs were recorded. Multivariate logistic regressions were also completed by comparing those who experienced multisystem irAEs, single irAE, and no irAE.

**Results**

A total of 123 patients were included in this study. The median age was 59 years, with 69% of patients being male. A total of 72 out of 123 patients (59%) had melanoma, 50 out of 123 (41%) had renal cell carcinoma (RCC), and 1 out of 123 (1%) had breast cancer. At least one irAE was observed in 72% of patients, and most irAEs occurred during the combination phase (89% of first irAE, 59% of second irAE, 75% of third irAE, and 50% of fourth irAE). Multisystem irAEs were observed in 40% of the overall cohort. The most common irAE type was dermatitis (22%), followed by colitis (19%), hepatitis (17%), and thyroiditis (15%). It was found that 30 out of 89 patients (34%) who experienced irAE(s) required hospitalization for treatment of irAE(s).

**Conclusions**

From our single-center cohort study, there appeared to be a higher than previously quoted percentage of patients who experienced irAEs after receiving ipilimumab and nivolumab, with the majority of them developing multisystem irAEs. Many patients experienced debilitating associated symptoms and hospital admissions that were related to irAEs. It is important for both the counselling and consent of patients, and for patient education, to discuss the possibility of multiple irAEs prior to dual immunotherapy initiation.

## 20. Poster Presentation

**Improving the Frequency and Documentation of Goals of Care Conversations by Medical Oncologists at the Ottawa Hospital**
Julian Surujballi and Joanna Gotfrit
The Ottawa Hospital Cancer Centre, University of Ottawa, Ottawa, ON, Canada

**Objective**

This quality improvement project aims to assess and improve the rates of goals of care discussion (GOCD) documentation using an electronic documentation template for medical oncologists and nurses at The Ottawa Hospital Cancer Centre.

**Methods**

The primary intervention was in the form of the creation and distribution of a documentation template in the Epic electronic health record. The secondary intervention was in the form of education sessions for medical oncologists and nurses regarding holding GOCDs and using the template.

The rate of documented GOCDs within 60 days of consultation to medical oncology was assessed through a retrospective chart review. The rates of GOCDs were assessed for seven days before and after the education sessions. The results were assessed based on the treatment intent and by an individual oncologist. A survey assessing barriers to GOCDs was administered to medical oncologists after the evaluation period.

**Results**

During the assessment period, 88 and 111 charts were reviewed before and after the intervention, respectively. The rates of documented GOCDs in the overall population were not significantly different before and after the intervention (0.24 vs. 0.22, *p* = 0.71). The GOCD rates based on treatment intent were as follows: palliative (0.35 vs. 0.26, *p* = 0.16), adjuvant (0.18 vs. 0.20, *p* = 0.77), curative (0.18 vs. 0.25, *p* = 0.25), and neoadjuvant (0.18 vs. 0.25, *p* = < 0.01). Significant variations in the baseline documented GOCD rates were observed between providers (median 0.17, SD 0.26, minimum 0, maximum 0.67), which persisted after the intervention. Out of the documented GOCDs, 69% occurred at the initial visit. No GOCDs were documented by nurses.

The barrier survey response rate was 42.3% (*n* = 11). The top barriers that were identified were being unaware of the template (*n* = 4) and forgetting to use the template (*n* = 3).

**Conclusions**

Creating a documentation template and providing education did not significantly increase the rates of documented GOCDs among medical oncologists at The Ottawa Hospital. Future directions include further education and engaging in patient GOCDs outside of clinic visits.

## 21. Poster Presentation

**The Con Experience in Advanced Ovarian Cancer: Are We Meeting the Standard?**
Kelly Li ^1^, Samuel Leung ^2,3^, Aline Talhouk ^3,4^ and Anna Tinker ^1,3^

1. Division of Medical Oncology, BC Cancer Agency, Vancouver, BC
2. BC Cancer Research Institute, Vancouver, BC
3. Gynecological Cancer Outcomes Unit, BC Cancer Agency Vancouver, BC
4. Division of Gynecological Oncology, BC Cancer Agency Vancouver, BC

**Background**

Management of advanced ovarian cancer requires a multidisciplinary team. The community oncology network (CON) in British Columbia (BC) connects local health centers with the BC cancer agency, enabling patients to receive cancer care that is close to home. The CON is defined by a Tiers of Service framework, ranging from tier 1 (primary care) to 6 (subspecialized province-wide consultations). We aim to identify whether patients undergoing treatment for advanced ovarian cancer at tier 3/4 CON centers received the same care compared with those at tier 5/6 centers.

**Methods**

Patients with advanced, high-grade serous ovarian cancer diagnosed from 1 January 2017 to 30 June 2019 were identified. A total of 30 patients from tier 3/4 CON centers and 30 patients from tier 5/6 centers with follow-up data were selected. Information on the diagnostic and treatment details was collected and analyzed.

**Results**

It was found that 20% of patients from tier 3/4 CON centers had cytology only for diagnosis compared with 33.3% of patients from tier 5/6 centers. The results showed that 70% of patients from tier 3/4 centers received pre-operative chemotherapy (POC) and 63.3% of patients from tier 5/6 centers received POC. For both tier 3/4 and tier 5/6 centers, most patients (83.3%) had a surgical consultation with a gynecological oncologist and 70% had debulking surgery. The median number of cycles of POC received by patients prior to surgical debulking was five among tier 3/4 CON patients and four among those from tier 5/6 centers. It was discovered that 60% of patients treated in tier 3/4 and 63.3% from tier 5/6 centers received adjuvant chemotherapy. The referral rates to the Hereditary Cancer Program was high (81.7%) among patients from both groups.

**Conclusions**

In the pre-COVID era, BC patients with advanced ovarian cancer that were treated at tier 3/4 CON centers received care that was similar to those treated at tier 5/6 centers. Tier 3/4 patients received more cycles of POC, possibly reflecting a delay in coordinating surgical care. 

## 22. Virtual Poster Presentation

**A Contemporary Assessment of the Landscape of Canadian Undergraduate Medical Education (Ugme) Oncology Training**
Sumiya Lodhi ^1^, William Phillips ^2^, Jerry Maniate ^2^, Craig Campbell ^2^, Anna Wilkinson ^3^, Sandeep Sehdev ^4^

1. Department of Medicine, University of Ottawa, Ottawa, Ontario, Canada
2. Department of Medicine, The Ottawa Hospital, Ottawa, Ontario, Canada
3. Department of Family Medicine, The University of Ottawa, Ontario, Canada
4. The Ottawa Hospital Cancer Centre, Ottawa, ON, Canada

**Objective**

This initiative aims to identify specific gaps in undergraduate medical education (UGME) curricula to guide future curriculum improvement.

**Methods**

A mixed-method model was employed using initial surveys of educational stakeholders, including medical students, recently graduated family physicians, and UGME leadership. Among medical students, only executive members of the Canadian student councils were asked to participate. Surveys were designed to assess oncology training structure, methods, and formal teaching time for core oncology competencies. Participants were recruited via email, and surveys were distributed using Google Forms.

**Results**

We received 14 responses from UGME leadership, 29 from medical students and 17 from family physicians. UGME leaders from 13 of 17 Canadian institutions and medical students from 8 of 17 Canadian institutions responded. Overall, 13 (45%) medical students did not feel prepared for cancer care. Students felt the least prepared for managing survivorship and complications of active cancer/cancer therapies, which were allotted at < 5 h as reported by 10 (71.4%) people from UGME leadership. More than 50% of family physicians reported undergraduate and residency training as below average or unsatisfactory. Family physicians identified cancer screening, survivorship, and communication as the most relevant competencies to their practice.

**Conclusions**

Despite cancer being the leading cause of death in Canada, medical graduates do not feel prepared for cancer care. These results identify the survivorship and management of cancer and cancer treatment complications as areas of deficiency that would benefit from more comprehensive teaching and exposure in UGME training. 

## 23. Virtual Poster Presentation

**Locally Advanced Rectal Cancer Referrals at the Ottawa Hospital Cancer Centre: An Audit**
**Victor Lo and Joanna Gotfrit**
Division of Medical Oncology, Department of Medicine, University of Ottawa, Ottawa, Ontario

**Objective**

Locally advanced rectal cancer patients require multidisciplinary care for treatment planning. We determined the proportion of patients who had all necessary consultations and investigations prior to their medical oncology consultation, and we planned to assess the logistical, system, and clinical factors that may be associated with increased time from diagnosis to treatment initiation.

**Methods**

We conducted a retrospective chart review of adult patients that were referred to The Ottawa Hospital Cancer Centre with locally advanced rectal adenocarcinoma as observed in a consultation performed by a surgical, medical, and radiation oncologist between January 2019 and December 2022.

**Results**

Data collection and analysis are ongoing. Out of 60 patients, the median age was 63 years (range: 22–83); a total of 36 (60%) patients were male. The median time from diagnosis to treatment was 57 days (range: 23–108). All patients underwent MRI imaging, with 58 (97%) also completing an abdomen–pelvis CT scan and 51 (85%) completing a chest CT scan. A total of 49 (82%) patients met the criteria for high-risk disease as per the RAPIDO trial, and 11 (18%) patients required presentation at multidisciplinary case conferences (MCCs). Surgical oncology consultation preceded medical oncology consultation in 34 (57%) patients, and 26 (43%) patients required an additional appointment with their medical oncologist prior to treatment initiation (range: 1–2 additional appointments). The most common initial treatment plans included non-operative management (35%), total neoadjuvant therapy with planned resection (22%), long-course chemoradiation (23%), short-course radiation, (13%), and upfront resection (5%).

**Conclusions**

Many patients did not have a surgical oncology opinion at the time of the medical oncology consultation, and almost half of all patients required additional appointments with medical oncology prior to treatment initiation. Future analysis aims to determine whether the initial treatment approach, order of specialist consultations, availability of staging investigations at time of medical oncology consultation, and need for presentation at MCCs are associated with increased time-to-treatment initiation.

## 24. Virtual Poster Presentation

**Eligibility and Workload Impact of Introduction of Adjuvant Nivolumab in Patients with Resected Esophageal and Gastroesophageal Junction (ESO/GEJ) Cancer**
Tae Hoon Lee ^1^ and Sharlene Gill ^1,2^

1. Department of Medicine, University of British Columbia, Vancouver, British Columbia
2. BC Cancer, Vancouver, British Columbia

**Objectives**

The CheckMate (CM) 577 study demonstrated the benefit of 12 months of additional therapy with adjuvant nivolumab in patients with pathologic residual disease following neoadjuvant chemoradiation for ESO/GEJ cancer. Nivolumab is now funded in BC as per the *GIAJNIV* protocol. This BC Cancer retrospective study reviews the real-world eligibility and potential resource implications that are associated with the therapy.

**Methods**

An REB-approved chart review was conducted for patients who underwent *CROSS* chemoradiation at BC Cancer from January 2016 to December 2020. Patient eligibility was determined by the identification of at least ypT1 or ypN1, which was assessed as per the CM577 and *GIAJNIV* criteria. The resource impact of nivolumab was assessed by projecting the number of MD visits, chemotherapy visits, and anticipated G3/4 serious toxicity events as per the CM577 study.

**Results**

A total of 677 patients were identified: 63% were esophageal and 37% were GEJ, with 74% having adenocarcinoma and 25% having squamous cell carcinoma histology. A total of 460 patients underwent resection, with 365 patients (79%) having pathologic residual disease. By the CM577 criteria, *n* = 249 (68%) were eligible for adjuvant nivolumab, while *n* = 321 (88%) were eligible as per the *GIAJNIV* criteria. In BC, with conservative assumptions, this translates into an estimated 60 patients/year being eligible for adjuvant nivolumab, resulting in 768 additional new chemotherapy visits and a potentially equal number of MD visits, which translates into 254 additional MD work hours annually. Based on a 34% G3/4 toxicity rate, an estimated 20 patients/year would result in a serious toxicity event that requires medical intervention.

**Conclusions**

Adjuvant nivolumab is an important new treatment option for patients with resected ESO/GEJ cancer. Our findings suggest that most patients (88%) with resected ESO/GEJ cancer and residual disease would be eligible for 12 months of adjuvant nivolumab. In addition to treatments costs, the additional oncologist workload impact should be considered in the provincial implementation of therapies for new indications.

## 25. Virtual Poster Presentation

**Real-World Experience of Cemiplimab in the Treatment of Refractory Locally Advanced and Metastatic Cutaneous Squamous Cell Carcinoma**
Scott Strum ^1^, Seth Climans ^2^, Victoria Purcell ^3^, Morgan Black ^1^ and Scott Ernst ^1^

1. Department of Oncology, Western University, London, Ontario, Canada
2. Department of Clinical Neurological Sciences, Western University, London, Ontario, Canada
3. Schulich School of Medicine and Dentistry, Western University, London, Ontario, Canada

**Background**

Cutaneous squamous cell carcinoma (cSCC) is the second most common non-melanoma skin cancer in Canada. However, to date, few real-world reports exist on the treatment of refractory locally advanced (non-metastatic cSCC no longer amenable to surgical intervention or radiation therapy) or metastatic cSCC using cemiplimab. The objective of this study was to characterize the demographic and clinical outcomes of these patients in a real-world setting.

**Methods**

A retrospective analysis was performed on adult patients with refractory locally advanced (LA) and metastatic cSCC that was treated using cemiplimab at the London Regional Cancer Program in Ontario, Canada. The patient demographics and treatment characteristics were reported, as well as the Kaplan–Meier estimates of progression-free survival (PFS) and overall survival (OS).

**Results**

Forty patients were included in this study. A total of 15 (38%) had an LA disease at the time of cemiplimab treatment, and 25 (62%) had a metastatic disease. The median treatment duration was 5.2 months (IQR 1.6 months–10.3 months). The Kaplan–Meier analysis revealed that the median OS was not reached (NR) for LA patients but was 10 months (95% CI 3.25 months-NR) for metastatic patients. The median PFS was 25.3 months (95% CI 7.29 months-NR) for LA patients and 7.1 months (95% CI 3.02 months-NR) for metastatic patients. The estimated probability of OS at 12 months for all patients was 58.6% (95% CI 43.2%–79.4%), and the estimated probability of PFS at 12 months was 45.3% (95% CI 30.4%–67.5%). Reasons for treatment discontinuation were death from any cause (44%), disease progression (22%), cemiplimab side effects (4%), and other causes (30%; comorbidities, treatment breaks).

**Discussion**

The estimated 12-month OS and PFS values were lower than the corresponding pivotal phase I and II clinical trials. However, the toxicity was tolerable, and no deaths were attributable to cemiplimab. Thus, cemiplimab is a safe and effective therapy in patients with refractory locally advanced and metastatic cSCC disease.

## 26. Virtual Poster Presentation

**Hepatocellular Carcinoma (HCC) in Alberta, Canada: A Retrospective Database Analysis to Understand Treatment Patterns and Outcomes in Intermediate and Advanced Unresectable HCC**
Chloe Lim ^1^, Carla Pires Amaro ^2^, Philip Ding ^2^, Winson Cheung ^2^, Iqra Syed ^3^, YongJin Kim ^3^, Derek Clouthier ^3^, Sharon Wang ^3^, Cal Shephard ^3^, Vincent Tam ^2^

1. Internal Medicine Residency Program, University of Calgary, AB
2. Department of Oncology, University of Calgary, AB,
3. AstraZeneca Canada, Mississauga, ON;

**Background**

With the emergence of new systemic therapies, there has been a substantial change in the treatment of HCC. The aim of this study was to assess the real-world data regarding treatment patterns and outcomes in Canadian HCC patients with an intermediate (BCLC-B) and advanced (BCLC-C) stage disease who received systemic treatments.

**Methods**

All BCLC-B and BCLC-C HCC patients who received at least one dose of systemic therapy between 1 January 2008 and 31 December 2020 in Alberta, Canada, were included. The patient characteristics, treatment patterns, overall survival (OS), progression-free survival (PFS), clinician-assessed response rates (RR), and reasons for treatment discontinuation were retrospectively analyzed across both BCLC stages.

**Results**

Of total 321 patients included, 33 (10%) were BCLC-B and 288 (90%) were BCLC-C. The demographic and clinical characteristics were as follows for the BCLC-B and BCLC-C groups, respectively: The median age was 65 and 63 years; out of the two groups, 91% and 80% were male, and 9% and 20% were of East Asian ethnicity; most patients were ECOG 0–1 (94% and 85%) and Child-Pugh A (82% and 85%); in both groups, 97% were albumin-bilirubin (ALBI) grade 1 or 2 before the start of first-line treatment; common causes of liver disease were hepatitis C (33% and 42%), alcohol (36% and 25%), and hepatitis B (15% and 24%); prior treatments included TACE (34% and 30%), TARE (6% and 8%), liver resection (24% and 17%), and SBRT (15% and 4%). Table 1 outlines the systemic treatments received and the outcomes by BCLC stage. The median follow-up was 13.5 months and 10 months for the BCLC-B and BCLC-C groups, respectively. The median treatment duration of systemic therapy for all patients was 4 months.

**Conclusions**

Although this study was not powered for a formal comparison, the systemic treatment patterns and outcomes of BCLC-B and C HCC patients seemed comparable, with a possible trend towards better PFS and OS in BCLC-B patients.

**Table 1:** Outlines the systemic treatments received and the outcomes by BCLC stage.

	Overall *n* = 321	Intermediate Stage (BCLC B) *n* = 33	Advanced Stage (BCLC C) *n* = 288	*p* Value
First-line treatment - sorafenib - lenvatinib - atezo+bev	79% 14% 3%	76% 21% 3%	80% 13% 4%	0.72
RR	19%	13%	19%	0.25
Median PFS (95% CI), months	4.4 (3.8–5.6)	7.4 (5.1–15.9)	4.2 (3.5–5.3)	0.08
Median OS (95% CI), months	11.1 (9.9–12.9)	13.5 (11.6–25)	10.9 (9.6–2.6)	0.32
Reason for discontinuation - Progression - Toxicity - Patient Choice - Death	58% 28% 6% 7%	57% 27% 13% 3%	59% 29% 5% 8%	0.35

## 27. Virtual Poster Presentation

**Assessing for Optimal Utilization of the Medical Oncology Inpatient Unit at the Ottawa Hospital**
Julian Surujballi and Paul Wheatley-Price
The Ottawa Hospital Cancer Centre, University of Ottawa, Ottawa, ON, Canada

**Objective**

Inpatient medical oncology units face growing capacity pressures as systemic therapy becomes increasingly complex. This quality improvement initiative assesses adherence to local admission criteria for the inpatient medical oncology ward at The Ottawa Hospital.

**Methods**

In phase 1, medical oncology inpatient admissions during January 2020 were retrospectively assessed for adherence to local admission guidelines. The reasons for admission were additionally reviewed by two medical oncologists to assess if cases were most appropriately primarily cared for by a medical oncologist versus another specialist.

In phase 2, on-call personnel were asked to log and rate the appropriateness of all referrals from the emergency department from February to November 2020.

**Results**

In phase 1, 80% of admissions were referred through the emergency department and 20% from direct admissions or transfers. We report that 59% of admissions were adherent to the referral guidelines, 26% were non-adherent, and 15% were unclear. When assessed by medical oncologists, 63% of admissions were thought to be appropriate, 14% were best cared for by another specialist, and 23% required collaborative care.

In phase 2, 71 referrals were recorded, which mostly occurred before June 2020. Of these referrals, 62% were deemed appropriate for medical oncology admission versus 38% being deemed as inappropriate. The reasons for deeming a referral inappropriate included hemodynamic instability (13.2%), respiratory distress (2.6%), insufficient workup (15.8%), requiring admission for a non-oncologic reason (15.8%), or requiring a non-oncologic specialty (52.6%).

**Conclusions**

There exists an opportunity for the optimization of medical oncology inpatient service utilization, although results are confounded by competing inpatient pressures that are caused by the COVID-19 pandemic. With medical oncology being a primarily outpatient specialty, reserving the oncology inpatient unit for patients that require specialized oncology care could allow for resources to be diverted to the outpatient setting. As oncology care becomes more complex, a multidisciplinary approach to inpatient cancer care may be required.

## 28. Virtual Poster Presentation

**A Patient Survey Evaluating COVID-19-Induced Changes in the Follow-up of Patients with EBC: Opportunities for Evidence-Based Practice?**
Ana-Alicia Beltran-Bless ^1^, Gail Larocque ^2^, Muriel Brackstone ^3^, Angel Arnaout ^4^, Jean-Michel Caudrelier ^5^, Denise Boone ^2^, Parvaneh Fallah ^1^, Terry Ng ^1,6^, Peter Cross ^5^, Nasser Alqahtani ^1^, John Hilton ^1,2^, Lisa Vandermeer ^6^, Gregory Pond ^7^, Mark Clemons ^1,2,6^

1. University of Ottawa, Ottawa, Ontario
2. The Ottawa Hospital Cancer Centre, Ottawa, Ontario
3. Department of Surgery, London Health Sciences Centre, London, Ontario
4. Department of Surgery, The Ottawa Hospital Cancer Centre, Ottawa
5. Department of Radiation Medicine, The Ottawa Hospital Cancer Centre, Ottawa, Ontario
6. Cancer Therapeutics Program, The Ottawa Hospital Research Institute, Ottawa, Ontario
7. Department of Oncology, McMaster University, Hamilton, Ontario

**Purpose**

Following the completion of early breast cancer (EBC) treatment, guidelines recommend routine in-person follow-up with a physical examination. The adoption of virtual visits during the COVID-19 pandemic resulted in major changes in practice. A patient survey was conducted to understand the current practices, perspectives, and desires of a follow-up.

**Methods**

EBC patients who have completed the acute treatment phase (i.e., surgery, chemotherapy, and radiation) were approached. The survey evaluated the current follow-up schedule, perceptions about follow-up, as well as interest in clinical trials.

**Results**

Of the 402 patients who were approached, 239 responses were obtained (response rate 59%). The median age of participants was 62.5 years (range 25–86 years). Routine follow-up appointments were most often conducted by patients’ medical (*n* = 147/244, 60%) or radiation oncologist (*n* = 130/244, 53%). Patients were often followed by multiple healthcare providers (mean 1.7). Breast examinations were conducted every 6 months (*n* = 110/236, 46%) or annually (*n* = 106/236, 44%).

Regularly scheduled follow-up was important to monitor for local recurrence, distant recurrence, manage the side effects of treatment, and to provide psychological support. Most patients were satisfied with their follow-up frequency (satisfaction score 8.26/10) and would worry more about their cancer if there were no visits (*n* = 225/247,91%). Patients felt that regular follow-up would detect recurrent cancer earlier (*n* = 214/255, 96%) and would help them live longer (*n* = 218/249, 88%). While most patients felt that their medical oncologist was the most suited for providing follow-up, 55% of patients felt comfortable being followed by their family physician. The COVID-19 pandemic reduced the number of in-person breast examinations for 54% of patients (*n* = 63/117). Many patients were concerned that this would lead to a later detection of local (*n* = 29/63, 46%) and distant recurrence (*n* = 25/63, 40%).

**Conclusions**

Despite evidence showing little impact on in-person assessment and the move to virtual care on the detection of recurrence, patients continue to place importance on regularly scheduled in-person follow-ups.

## 29. Virtual Poster Presentation

**Evolving Strategies for The Routine Follow-Up of Patients With Early Breast Cancer and the Impact of COVID-19: A Survey of Healthcare Providers**
Ana-Alicia Beltran-Bless ^1^, Gail Larocque ^2^, Angel Arnaout ^3^, Jean-Michel Caudrelier ^4^, John Hilton ^1,2^, Nasser Alqahtani ^1^, Lisa Vandermeer ^5^, Gregory Pond ^6^, Mark Clemons ^1,2,5^

1. University of Ottawa, Ottawa, Ontario
2. The Ottawa Hospital Cancer Centre, Ottawa, Ontario
3. Department of Surgery, The Ottawa Hospital Cancer Centre, Ottawa, Ontario
4. Department of Radiation Medicine, The Ottawa Hospital Cancer Centre, Ottawa, Ontario
5. Cancer Therapeutics Program, The Ottawa Hospital Research Institute, Ottawa, Ontario
6. Department of Oncology, McMaster University, Hamilton, Ontario

**Purpose**

The COVID-19 pandemic resulted in a rapid change in routine follow-ups for early breast cancer (EBC). A survey was performed to explore the perceptions of healthcare providers (HCPs) around the current practices and goals of follow-ups.

**Methods**

Canadian HCPs who treat EBC participated in an anonymous electronic survey. Participants provided perspectives on follow-ups, current practices regarding in-person and virtual visits, and interest in clinical trials assessing follow-up strategies.

**Results**

Responses were received from 73 HCPs, including medical (*n* = 41/73,56%), radiation (*n* = 13/73,18%), and surgical oncologists (*n* = 13/73,18%). Thirty-four percent (*n* = 25/73) of HCPs did not perform routine follow-ups. Of the 48 (*n* = 48/73,66%) participants who conducted in-person follow-ups, the follow-ups were typically conducted once every six months for years 1–3, yearly until year 5, and then on demand. Common reasons for follow-up visits were the assessment of symptoms from endocrine therapy and for the detection of recurrent disease. HCPs felt that routine follow-ups with physical examinations resulted in the earlier detection of local (*n*= 16/48,33%) and distant metastases (*n*= 6/48,12.5%). While 48% of HCPs felt that the transition to virtual visits would neither impact local nor distant recurrence or overall survival, 42% thought it would lead to a later detection of local recurrence and 33% thought it would lead to a later detection of distant recurrences. Sixty-nine percent (*n* = 33/48) will continue to follow patients with a combination of in-person and virtual appointments. Most respondents agreed that follow-up should be more individualized and risk-adapted (*n* = 42/48,87.5%). Most (62%, *n* = 29/47) HCPs expressed interest in performing trials assessing good follow-up strategies.

**Conclusion**

Virtual care will remain an integral part of routine follow-ups. The effects of this on a range of patient outcomes should be explored in future trials.

## 30. Virtual Poster Presentation

**Treatment Patterns of Pancreatic Neuroendocrine Tumor (pNET) Patients at the London Regional Cancer Program (LRCP) and the Ottawa Hospital Cancer Centre (TOHCC)**
Gautham Nair ^1^, Stephen Welch ^2^, David Laidley ^2^, Robin Sachdeva ^3^, Rachel Goodwin ^4^, Macyn Leung ^5^, Tim Asmis ^5^, William Phillips ^6^, Morgan Black ^3^, Michael Vickers ^4^, Horia Marginean ^6^ and Elena Tsvetkova ^2^

1. Western University, London, ON
2. London Health Sciences Centre, London, ON
3. Lawson Health Research Institute, London, ON
4. The Ottawa Hospital, Ottawa, ON
5. The Ottawa Hospital Research Institute, Ottawa, ON
6. Department of Medicine, University of Ottawa, Ottawa, ON

**Objective**

To determine the treatment patterns of pNET patients managed at the LRCP and TOHCC.

**Methods**

This is a multicenter retrospective cohort study involving LRCP and TOHCC. Patients with histologically confirmed pNETs between January 2010 – June 2021 over the age 18 were included. Demographic, clinical and cancer treatments were collected. Descriptive analysis was performed on the data collected to identify the local and systemic therapies used.

**Results**

In total 192 pNET patients’ charts were reviewed, 40% treated at LRCP; 42% female. Most common pNET locations were the pancreatic head (34% ) and tail (45%) of the pancreas. 53% presented with stage IV disease and 21% with stage II. 28% were grade 1, 36% grade 2, and 8% grade 3 with the remainder either being unknown or having no pathology performed for the disease. 

Local treatments included surgery for the primary tumour in 56% of patients, with 40% having curative intent (Table 1). Surgery for metastasis occurred in 16% of patients and 63% of surgeries were in the liver. Local regional ablative treatments were used in 16%, 99% of ablations targeted metastases, and radiation was used in 15% of patients. The percentage of patients treated with systemic therapy included: 34% octreotide, 30% lanreotide, 30% chemotherapy, 29% PRRT, and 22% targeted therapy. The majority of patients undergoing systemic therapy had established metastases and progressive disease prior to treatment.

**Conclusions**

In this real-world database, 53% of patients present with non-curative disease and 15% of pNETs undergo metasetecomy, while embolization is not a common modality used. The most common systemic treatment used was somatostatin analogues followed by chemotherapy, PRRT and targeted therapy. This is one of the first cross centre, patient-level, Ontario databases that report the common treatment modalities used for pNETs. Further analysis will assess how treatments choices vary according to tumour grade.

**Table 1:** Summary of the treatment modalities used at LRCP and TOHCC for pNET patients.

Treatment Modality	Percentage of Patients Receiving Treatment (%)	Notes
Surgery for Primary Tumor	56	40% curative intent
Octreotide	34	66% had progressive disease and 82% had metastases at start
Lanreotide	30	77% had progressive disease and 75% had metastases at start
Chemotherapy	30	94% had progressive disease and 91% have metastases at start. 45% of treatments ceased due to disease progression. 18% of treatments were completed.
PRRT	29	Median number of cycles was 4. Of all treatment instances 80 % had progressive disease and 96% had metastases at start. 77% were completed.
Targeted Therapy	22	85% had progressive disease and 89% had metastases at start
Surgery for Metastases	16	63% targeted the liver. 31% had curative intent.
Locoregional Ablative Therapy	16	99% of ablations targeted metastases.
Radiation Therapy	15	93% of treatments were done with non-curative intent. 100% had progressive disease and 93% had metastases at start.

## 31. Virtual Poster Presentation

**A Retrospective Analysis of the Diagnosis of Gastroenteropancreatic Neuroendocrine Tumors (GEP-NETs) at the Ottawa Hospital Cancer Centre (TOHCC) and the Impact of COVID-19 on Diagnosis**
Mohammad Alrehaili ^1,2^, William Phillips ^3^, Timothy Asmis ^1,2,4^, Michael Vickers ^1,2,4^, Horia Marginean ^4^, Rachel Goodwin ^1,2,4^

1. University of Ottawa Division of Medical Oncology, Ottawa, ON, Canada
2. The Ottawa Hospital Cancer Centre, Ottawa, ON, Canada
3. University of Ottawa Department of Medicine, Ottawa, ON, Canada
4. The Ottawa Hospital Research Institute, Ottawa, ON, Canada

**Introduction**

The incidence of NETs is rising. Our objective was to assess the trends of GEP-NETs diagnosis (June 2010 to June 2021) at TOHCC and to explore if early COVID-19 pandemic data impacted these trends.

**Methods**

This is a single-center retrospective chart review containing data that were collected from June 2010 to June 2021. We searched all databases, including OACIS/EPIC, PACS and OPIS, and found 647 GEP-NETs patients. A descriptive analysis was performed using frequencies and related percentages.

**Results**

Of the 647 patients with GEP-NETs, the small bowel was the most common primary location (*n* = 210 cases, 32.4%), followed by the pancreas (*n* = 118, 18.2%) and an unknown primary (*n* = 99, 15.3%). Most of the cases were classified as metastatic or locally advanced on initial presentation, and stage 1/2 was found in 158 cases (23.8%). Lower GI tumors were the most common disease among early-stage NETs (*n* = 88, 55.7%). There were 5 cases in 2010–2011, and the average number per year period was 5.5 until 2016–2017, after which time the number of cases increased to 10, 15, 11, and 13 during the last 4 year-periods. Regarding early stage pancreatic and upper GI NETs, the total number of cases was 52 (32.9%) and 18 (11.4%), respectively. The number of pancreatic cases was 4 in 2010–2011, and the average number per year period throughout the last 10 years was 4.7. Cases of upper GI tumors ranged between 1 and 3 per year period, with an average of 1.6 over the last decade.

**Discussion**

At our center, most GEP-NETs were presented in the advanced setting. There has been an increase in the incidence of early-stage disease. Disease detection for all GEP-NETs was consistent throughout the last decade except for lower GI cases, which have increased since mid-2017, perhaps reflecting the adoption of Ontario FIT testing. Despite endoscopy closures during the pandemic, the cases of GEP-NETs did not decrease.

## 32. Virtual Poster Presentation

**Multicenter Population-Level Analysis of Systemic Therapy Use in Metastatic or Recurrent Prostate Cancer**
Heba Mohamed ^1^, Grace Kim ^2^, Corin Macphail ^3^, Ishmam Bhuiyan ^4^, Taylor Sidhu ^4^, Amir Emami ^1^, Longlong Huang ^5^, Shaun Zheng Sun ^5^, Scott Tyldesley ^6^ and Jenny Ko ^1^

1. Medical Oncology, BC Cancer, Abbotsford, BC
2. Faculty of Medicine, University of British Columbia, Vancouver, BC
3. Department of Pediatrics, University of Alberta, Edmonton, AB
4. Faculty of Medicine, University of British Columbia, Vancouver, BC
5. Department of Mathematics and Statistics, University of Fraser Valley, Abbotsford, BC
6. Radiation oncology, BC Cancer, Vancouver, BC

**Background**

Treatment (tx) options for metastatic prostate cancer (mPC) have significantly advanced. We evaluated the real-world prescribing patterns and outcomes of new therapeutics.

**Methods**

We reviewed all consecutive patients (pts) diagnosed with PC between 2016 and 2017 in British Columbia; only pts with de novo metastatic or recurrence were included. We performed descriptive statistics and univariate/multivariate analyses to examine the overall survival (OS).

**Results**

Of *n* = 796 (de novo 69.6%; recurrence 30.4%) patients, 93.3% had metastasis by cutoff (Sep 1, 2022). The median age at diagnosis of mPC was 73 years (range 45–98 years). It was found that 99.2% of patients started ADT. An additional line of tx started with ADT in 35.4% of mCSPC and in 38.8% of mCRPC cases. Out of the patients, 18.7% tested for homologous repair defects (21 positive). Radiotherapy to the prostate for mPC was performed for 93 patients (11.6%). It was found that 307 (38.6%) patients received 1 line of tx; 181 (22.7%) received 2; and 128 (16.1%) received 3 or more. A total of 432 (54.3%) patients received ≥ 1 androgen receptor-axis-targeted therapy (ARAT). It was found that 59.5% of patients died (84.4% died from prostate cancer). Pts with ADT alone or one additional systemic tx had a better OS than those who had more tx lines (NR vs. 55.5 m vs. 21.0 m, *p* = 0.03). A longer OS was observed for pts with no or one ARAT than for those with multiple ARATs (NR vs. 48.5 m vs. 22.5 m, *p* = 0.009). No statistical difference in OSs was seen between pts who had chemotx vs. none or pts on trial vs. none. No other clinical factors had an independent effect on the OS. Pts with major cardiovascular/neurologic comorbidities received ARATs at a similar rate.

**Conclusions**

We observed a low uptake of early treatment intensification. Pts who required less switching to alternate systemic tx had a better OS. Different ARATs were prescribed at a similar rate to pts with various comorbidities regardless of the potential side effects.

